# Cognitive reserve index and long-term disability in patients with severe traumatic brain injury discharged from the intensive rehabilitation unit

**DOI:** 10.3389/fneur.2023.1106989

**Published:** 2023-05-05

**Authors:** Bahia Hakiki, Silvia Pancani, Anna Maria Romoli, Francesca Draghi, Daniela Maccanti, Andrea Mannini, Francesca Cecchi

**Affiliations:** ^1^IRCCS Fondazione Don Carlo Gnocchi, Florence, Italy; ^2^Department of Experimental and Clinical Medicine, University of Florence, Florence, Italy

**Keywords:** traumatic brain injury, cognitive reserve, functional status, rehabilitation, disability

## Abstract

**Objectives:**

The “cognitive reserve” (CR) theory posits that higher premorbid cognitive activities can mitigate the effects of brain damage. This study aimed to investigate the association between CR and long-term functional autonomy in patients surviving a severe traumatic brain injury (sTBI).

**Setting:**

Data were collected from the database of inpatients with severe acquired brain injury in a rehabilitation unit admitted from August 2012 to May 2020.

**Participants:**

Patients that had incurred an sTBI, aged 18+ years, completing the phone Glasgow Outcome Scale-Expanded at follow-up (pGOS-E) in absence of previous brain trauma or neurological disease, or cognitive disorders were included. Patients with severe brain injury from non-traumatic etiologies were not included in the study.

**Design:**

In this longitudinal study, all patients underwent a multidimensional assessment including the cognitive reserve index questionnaire (CRIq), the coma recovery scale-revised, the level of cognitive functioning, the Disability Rating Scale (DRS), and the Galveston Orientation and Amnesia Test at admission. At discharge, functional scales were administered again together with the Glasgow Outcome Scale. The pGOS-E was assessed at follow-up.

**Main measures:**

pGOS-E.

**Results:**

A total of 106 patients/caregivers underwent the pGOS-E after 5.8 [3.6] years from the event. Among them, 46 (43.4%) died after discharge, and 60 patients [men: 48 (80%); median age: 54 years; median time post-onset: 37 days; median education level: 10 years; median CRIq total score: 91] were included in the analysis exploring the association between pGOS-E and demographic data, cognitive reserve surrogates, and clinical variables at admission and discharge from the rehabilitation unit. A younger age (*B* = −0.035, *p* = 0.004) and a lower DRS category at discharge (*B* = −0.392, *p* = 0.029) were significantly related to a higher long-term functional autonomy in the multivariate analysis.

**Conclusion:**

Long-term functional autonomy was not influenced by CR as assessed through the educational level and the CRIq.

## 1. Introduction

Traumatic brain injury (TBI) is damage to the brain resulting from an external mechanical force causing a temporary or permanent impairment of brain function (1). In Europe, an estimated incidence rate of TBI is 235 persons per 100,000 population per year of which about 9% are severe (2). While in the US, the Centers for Disease Control and Prevention documented 2.53 million TBI-related emergency department visits in 2014. There were ~288,000 TBI-related hospitalizations and 56,800 TBI-related deaths (1). TBI is the first cause of disability in young adults worldwide leading to an important economic impact on national health systems (1). The functional and cognitive recovery after a TBI is characterized by high variability (3). This heterogeneity in outcome has been attributed partly to injury characteristics, such as the brain lesion dimension and characteristics (4) and severity (5). Based on the trauma severity, TBI may be distinguished as mild, moderate, or severe. In this context, severe TBI (sTBI) is related to a pathological event of a non-congenital, perinatal, or degenerative nature determining a coma condition, with Glasgow Coma Scale score-acute phase (GCS) between 3 and 8, lasting more than 24 h, presence of brain imaging abnormalities, and post-traumatic amnesia (PTA) >7 days. For their peculiarities, consisting essentially of a high care burden and/or the presence of a disorder of consciousness (DoC), patients surviving an sTBI are often transferred to a severe acquired brain injury rehabilitation unit (sABI-IRU) where a specific rehabilitation assessment protocol is applied (6). In fact, differently from other TBIs of minor severity, sTBI often leads to a coma or prolonged DoC that may persist or be followed by an emergence of consciousness. After consciousness recovery, patients often show a confusional state which is characterized by persistent dysfunction across multiple cognitive domains, behavioral dysregulation, symptom fluctuation, disorientation, and, hence, altered consciousness (7). Survivors of sTBI frequently suffer from several neurological symptoms leading to lifelong disability even though some patients achieve full recovery (8). In addition, recent studies have highlighted the higher risk of developing cognitive impairment or dementia after sTBI (9, 10).

The heterogeneity of recovery after a TBI has also been attributed to some patients' specificities (11), including their cognitive reserve. The theory of the “cognitive reserve” (CR) (12) has been proposed to explain individual differences in susceptibility to brain damage. It postulates that individual differences in the cognitive processes or neural networks underlying task performance enable some people to cope better with pathological brain damage and with normal aging brain changes (13). The theory of CR derives from research on aging and dementia (14) postulating that individual differences in the cognitive processes or neural networks allow some people to cope better with brain damage (14). In other words, the “reserve” can serve as a moderator between the pathology and its clinical expression. A differential classification of the “reserve” in brain reserve (the passive component of the reserve) and cognitive reserve (CR) (the active component of the reserve) has been established. The first, a quantitative component, consists of more neurons or more synapses that can be lost without reaching a threshold where clinical symptoms appear. In contrast, the concept of CR hypothesizes that the brain actively attempts to contrast brain changes by pre-existing cognitive processing approaches or by finding new compensatory strategies (10). The most accepted surrogate marker for CR is the level of educational attainment (15). However, several tools for simultaneous assessment of multiple proxies of CR (i.e., education; occupation; intellectual, physical, social, or leisure activities) have been developed (16).

Previous observations of CR have focused primarily on patients with evolving chronic neurodegenerative conditions such as Alzheimer's disease, HIV, or multiple sclerosis (17–19). Over the past decade, growing attention has been dedicated to this theory applied to the model of TBI. The protective effect of a higher CR on long-term neurocognitive profile, disability, and autonomy has been explored in patients experiencing a TBI of different severity and after different timings from the acute event (20–28). Nevertheless, the protective effect of CR in mitigating the effects of TBI does not find consensus among the studies published so far. This discrepancy could partly be explained by the inhomogeneity of the samples studied in terms of TBI severity. Thus, most of the previous studies tended to mix moderate and severe TBI patients in their analysis (18, 20–23), relaxing the criteria for defining sTBI. In Fraser and colleagues' study (24), the mean of the worse GCS score was 9.47 (SD 4.33); in Stewart et al. (23) and Schneider et al. (22), the inclusion criteria were GCS ≥12 and PTA >24 h.

The aim of this longitudinal follow-up study was to investigate the role of CR on long-term functional disability in patients with sTBI discharged from an sABI-IRU.

## 2. Materials and methods

A non-concurrent cohort study was conducted, following STROBE guidelines (29); the study was performed as an observational single-site analysis. The principles of the Declaration of Helsinki were followed, and the study was approved by the Institutional Ethics Committee (17505_oss).

### 2.1. Participants

In this observational longitudinal study, subjects were selected from a database of patients admitted to the sABI-IRU of the IRCCS-Don Gnocchi Foundation of Florence from August 2012 to May 2020 following an sABI and contacted by phone between September 2021 and April 2022 to perform the phone Glasgow Outcome Scale–Expanded (pGOS-E) (30, 31). Written consent was obtained from the legal guardians of all patients.

Inclusion criteria were as follows: (1) patients having an sTBI related to a pathological event of a non-congenital, perinatal, or degenerative nature determining a coma condition, with GCS between 3 and 8, lasting more than 24 h, presence of brain imaging abnormalities, and PTA >7 days, (2) patients of 18 years of age or more, (3) patients who have completed the follow-up phone interview, and (4) patients with the absence of previous brain trauma, neurological disease, or cognitive disorders.

### 2.2. Interdisciplinary functional assessment and rehabilitation intervention

Upon admission to the sABI-IRU, all patients underwent a multidimensional interdisciplinary assessment performed by a team of professionals (neurologist, internist, physiatrist, physiotherapist, speech therapist, nurse, and neuropsychologist). Demographic and clinical history data were recorded (age, sex, race, educational level, history of alcohol or drug abuse, psychiatric history, and time post-onset), and clinical scales were administered, the coma recovery scale-revised (CRS-R) (32), the level of cognitive functioning (LCF) (33), the Disability Rating Scale (DRS) (34), and the Galveston Orientation and Amnesia Test (GOAT) (35), to assess the presence and duration of PTA. At discharge, all the functional scales were administered again. The Glasgow Outcome Scale (GOS) (36) at discharge assessed by the medical staff was also recorded.

### 2.3. Cognitive reserve measures

To evaluate the preinjury CR, in addition to the educational level, the CR Index questionnaire (CRIq) (37) of the patient was administered to caregivers at admission. CRIq is a semi-structured interview addressed to a family member who knows the detailed patient history and consists of three parts: CRI-Education: level of education, the raw score is the sum of years of schooling and years of extra-scholastic training; CRI-working activity: years worked at distinct levels of occupation based on the cognitive input required and the level of responsibility, the raw score is the number of years worked in proportion to the cognitive commitment that each occupation requires; and CRI-leisure time: all activities normally done during a person's free time, the raw score is the sum of the years passed in leisure-time activities in proportion to the frequency of the activities.

### 2.4. Rehabilitation treatment, follow-up assessment, and outcome

Based on the individual's assessment, the individual rehabilitation project was planned by an interdisciplinary team of neurorehabilitation professionals delivering an average of 3 h of specific treatment per day. In addition, the pharmacologic interventions were planned according to the patient's needs. Discharge was planned and carried out upon the decision of the interdisciplinary team, including the patient's family and caregivers, in agreement with the local health authority, either when the patient reached a plateau or when the patient achieved a functional improvement that allowed home discharge or transfers to a less specialized intensive rehabilitation setting.

To evaluate the functional disability, the pGOS-E was carried out on the patients or their principal caregivers if they were unable to carry out the interview.

The primary outcome was to investigate the possible association between CR (assessed by educational level and CRIq score) and long-term functional outcomes assessed by the pGOS-E.

The secondary outcome was to identify predictors of higher long-term functioning both at admission and at discharge from the IRU.

### 2.5. Statistical analysis

The analyses were performed using SPSS 27.0 software (IBM SPSS, Chicago, Illinois, USA). Continuous data were first tested for normality using the Shapiro–Wilk test. As data did not meet the criteria for normality (*p* < 0.05), they were summarized through their median value, interquartile range (IQR), and minimum–maximum range. Categorical variables were summarized with their frequencies and percentages. Then, the association between functional disability at follow-up measured using the pGOS-E and the participants' characteristics was first tested by using univariate linear regression analyses. Variables that were shown to be associated with the pGOS-E score at follow-up with a *p*-value of at least <0.1 were introduced into a multivariate linear regression model. Two models were built: the first (Model A) considered demographic characteristics, the time between event and follow-up, and clinical data at admission; the second (Model B) considered demographic characteristics, the time between event and follow-up, and clinical data at discharge as independent variables. In both models, the pGOS-E score at follow-up was assumed as the dependent variable. In all analyses, a *p*-value of < 0.05 was considered statistically significant.

## 3. Results

**Figure 1:**
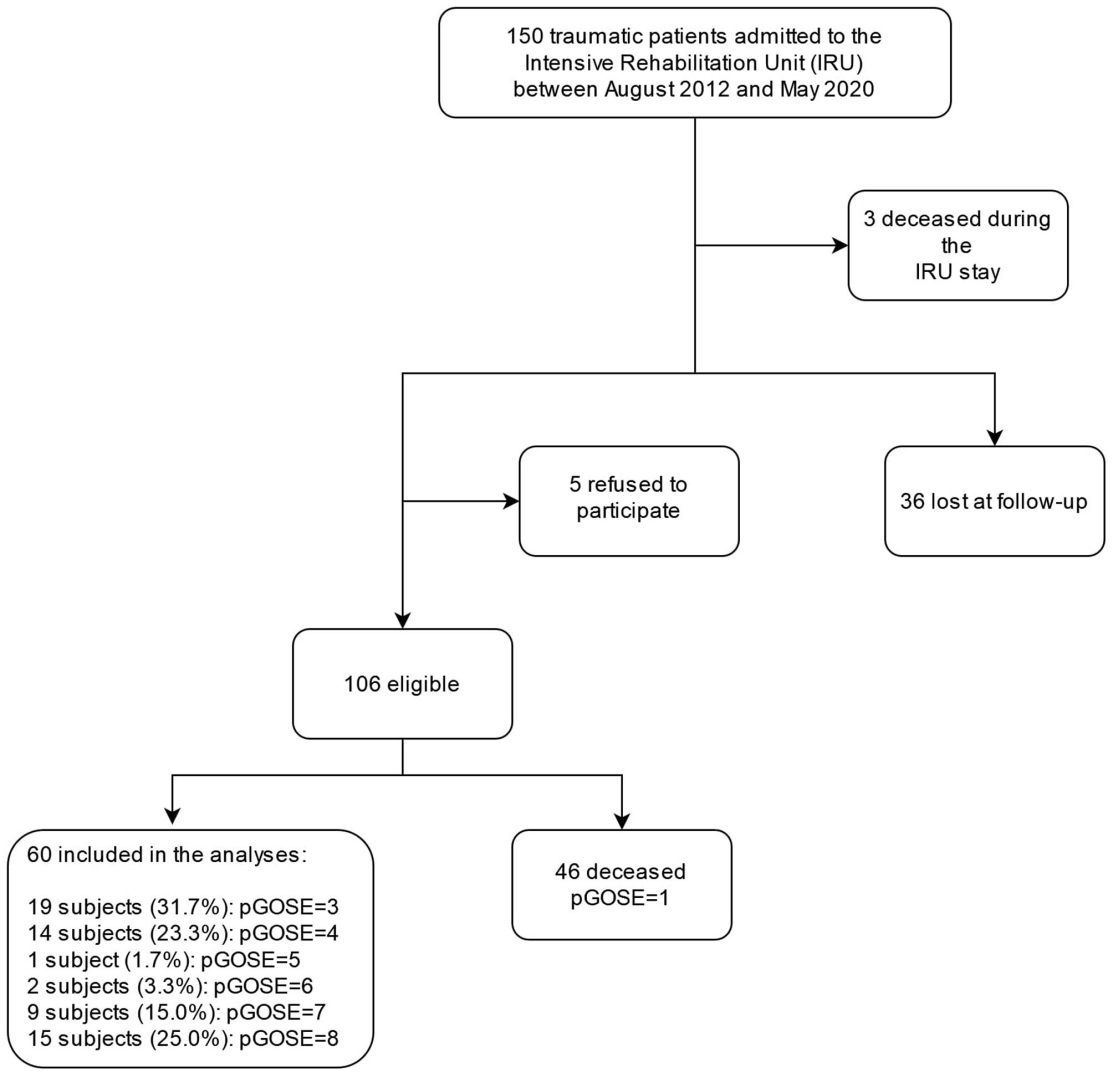
Flow chart of study population selection. pGOS-E, phone Glasgow Outcome Scale–Expanded.

A total of 150 patients with sTBI were admitted to the IRU between August 2012 and May 2020. Among them, three patients died during the IRU stay, 36 patients were lost at the follow-up, and five refused to participate in the phone interview. A total of 106 patients or their caregivers completed the phone interview at a median time from the event of 5.8 [IQR: 3.6] (range: 1.8–10.4) years. Among the 106 included patients, 46 (43.4%) died after their discharge from the IRU (Figure 1).

A total of 60 patients [men: 48 (80.0%); median age: 54, IQR: 34 years; median time post-onset (TPO): 37, IQR: 22 days; median education level: 10, IQR: 5 years; median CRIq total score: 91, IQR: 14] were included in the analysis. All included patients were native to Europe and were diagnosed in a state of a PTA at the admission time; thus, these variables were not included in the subsequent analysis. In total, 27 patients (45.0%) were in DoC at admission. At discharge from the IRU, after a median length of stay (LoS) of 109.5, IQR: 88 days, four patients (6.7%) were still in DoC, and 29 (48.3%) patients recovered from the PTA, with a median PTA duration of 75, IQR: 53 days. Demographic and clinical characteristics at admission and discharge are summarized in Table 1.

**Table 1:** Characteristics of the study sample at admission and discharge.

**Variables**	**Median [IQR]; (min–max) *N* (%)**
Age (years)	54 [34]; (18–85)
Sex (M)	48 (80.0%)
Education (years)	10 [5]; (3–23)
Native country	
Eastern Europe	2 (3.3%)
Western Europe	58 (96.7%)
TPO (days)	37 [22]; (13–501)
Time from event to follow-up (years)	5.8 [3.5]; (1.8–10.4)
LoS (days)	109.5 [88]; (22–324)
Abuse history (yes)	9 (15%)
Psychiatric history (yes)	7 (11.7%)
DRS at admission	7 [3]; (3–9)
LCF at admission	4 [1]; (1–7)
PTA at admission (yes)	60 (100%)
DRS at discharge	5 [2]; (0–8)
LCF at discharge	6 [2]; (2–8)
PTA at discharge (yes)	31 (51.7%)
CRI-education	91.5 [18]; (68–133)
CRI-working activity	94.5 [17]; (70–155)
CRI-leisure	95 [14]; (76–201)
CRI-tot	91 [14]; (76–167)
PTA duration (days)	75 [53]; (14–495)
pGOS-E at follow-up	4 [5]; (3–8)

IQR, interquartile range; TPO, time post-onset; LoS, length of stay; DRS, disability rating scale; LCF, levels of cognitive functioning; CRI, cognitive reserve index; PTA, post-traumatic amnesia; pGOS-E, phone glasgow outcome scale-expanded.

At follow-up, the median pGOS-E was 4, IQR was 5, and 27 patients presented moderate functional autonomy at follow-up (pGOS-E > 4).

To investigate a possible association between the CR surrogates and the pGOS-E at follow-up, univariate linear regression analyses were performed including pGOS-E as the dependent variable and the following independent variables: age, sex, educational level, CRIq, TPO, psychiatric and abuse history, DRS category at admission and discharge, CRS-R at admission and discharge, LCF at admission and discharge, LoS in the IRU, PTA recovery (yes/no) at discharge, the time between the event, and follow-up (Table 2). Neither education nor the CRIq was significantly associated with pGOS-E at follow-up (*p* = 0.259 and *p* = 0.326, respectively).

**Table 2:** Univariate linear regressions.

	**B**	**SE**	**Beta**	***t***	***p*-value**	**95%CI lower**	**95%CI upper**
Age (years)	−0.044	0.012	−0.425	−3.574	0.001	−0.069	−0.020
Sex (M)	1.208	0.665	0.232	1.817	0.074	−0.123	2.539
Education (years)	0.100	0.069	0.188	1.447	0.153	−0.038	0.238
TPO (days)	−0.003	0.003	−0.134	−1.032	0.306	−0.009	0.003
Time from event to follow-up (years)	0.277	0.126	0.277	2.196	0.032	0.024	0.529
Abuse history (yes)	0.529	0.763	0.091	0.694	0.490	−0.997	2.056
Psychiatric history (yes)	−1.054	0.840	−0.162	−1.254	0.215	−2.736	0.628
DRS at admission	−0.329	0.179	−0.235	−1.841	0.071	−0.686	0.029
LCF at admission	0.569	0.219	0.323	2.601	0.012	0.131	1.007
DRS at discharge	–0.558	0.139	–0.465	–4.002	<0.001	–0.837	–0.279
LCF at discharge	0.876	0.201	0.498	4.371	<0.001	0.475	1.278
PTA at discharge (yes)	–1.182	0.525	–0.284	–2.254	0.028	–2.233	–0.132
CRI-education	−0.028	0.019	−0.190	−1.470	0.147	−0.065	0.010
CRI-working activity	0.009	0.017	0.072	0.552	0.583	−0.024	0.043
CRI-leisure	−0.019	0.013	−0.187	−1.454	0.151	−0.046	0.007
CRI tot	−0.015	0.015	−0.129	−0.990	0.326	−0.046	0.016

Dependent variable: phone Glasgow Outcome Scale-Expanded (pGOS-E) score at follow-up; independent variables: demographic characteristics and clinical data at admission and discharge.

TPO, time post-onset; DRS, disability rating scale; LCF, levels of cognitive functioning; PTA, post-traumatic amnesia; LoS, length of stay; CRI, cognitive reserve index.

Variables associated with the pGOS-E score at follow-up with a *p*-value of at least < 0.1 were then included in a multivariate linear regression analysis to predict long-term autonomy both at admission to the IRU (Table 3, Model A) and discharge (Table 3, Model B). Time from event to follow-up was included in both models as a confounder due to its wide range that appeared to be associated with the pGOS-E at a univariate level.

**Table 3:** Multivariate linear regression models.

	**B**	**SE**	**Beta**	***t***	***p*-value**	**95%CI lower**	**95%CI upper**
**Model A**							
Age (years)	−0.037	0.012	−0.350	−3.034	0.004	−0.061	−0.012
Sex (M)	0.729	0.598	0.140	1.219	0.228	–0.470	1.928
DRS at admission	–0.148	0.167	–0.106	–0.885	0.380	–0.483	0.187
LCF at admission	0.322	0.214	0.183	1.510	0.137	–0.106	0.751
Time from event to follow-up (years)	0.176	0.117	0.176	1.504	0.138	–0.059	0.410
**Model B**							
Age (years)	−0.035	0.012	−0.338	−2.973	0.004	−0.059	−0.011
Sex (M)	0.833	0.554	0.160	1.502	0.139	–0.279	1.945
Time from event to follow-up (years)	0.092	0.110	0.092	0.830	0.410	–0.130	0.313
DRS at discharge	−0.392	0.175	−0.327	−2.241	0.029	−0.743	−0.041
LCF at discharge	0.208	0.314	0.118	0.663	0.510	–0.421	0.837
PTA at discharge (yes)	–0.211	0.565	–0.051	–0.373	0.711	–1.344	0.923

Dependent variable: phone Glasgow Outcome Scale–Expanded (pGOS-E) score at follow-up; independent variables: time from event to follow-up and demographic characteristics and clinical data at admission (Model A). Dependent variable: pGOS-E at follow-up; independent variables: time from event to follow-up and demographic characteristics and clinical data at discharge (Model B).

Model A: DRS, disability rating scale; LCF, levels of cognitive functioning.

R^2^ = 0.251.

Model B: DRS, disability rating scale; LCF, levels of cognitive functioning; LoS, length of stay; CRS, coma recovery scale; PTA, post-traumatic amnesia.

R^2^ = 0.363.

**Figure 2:**
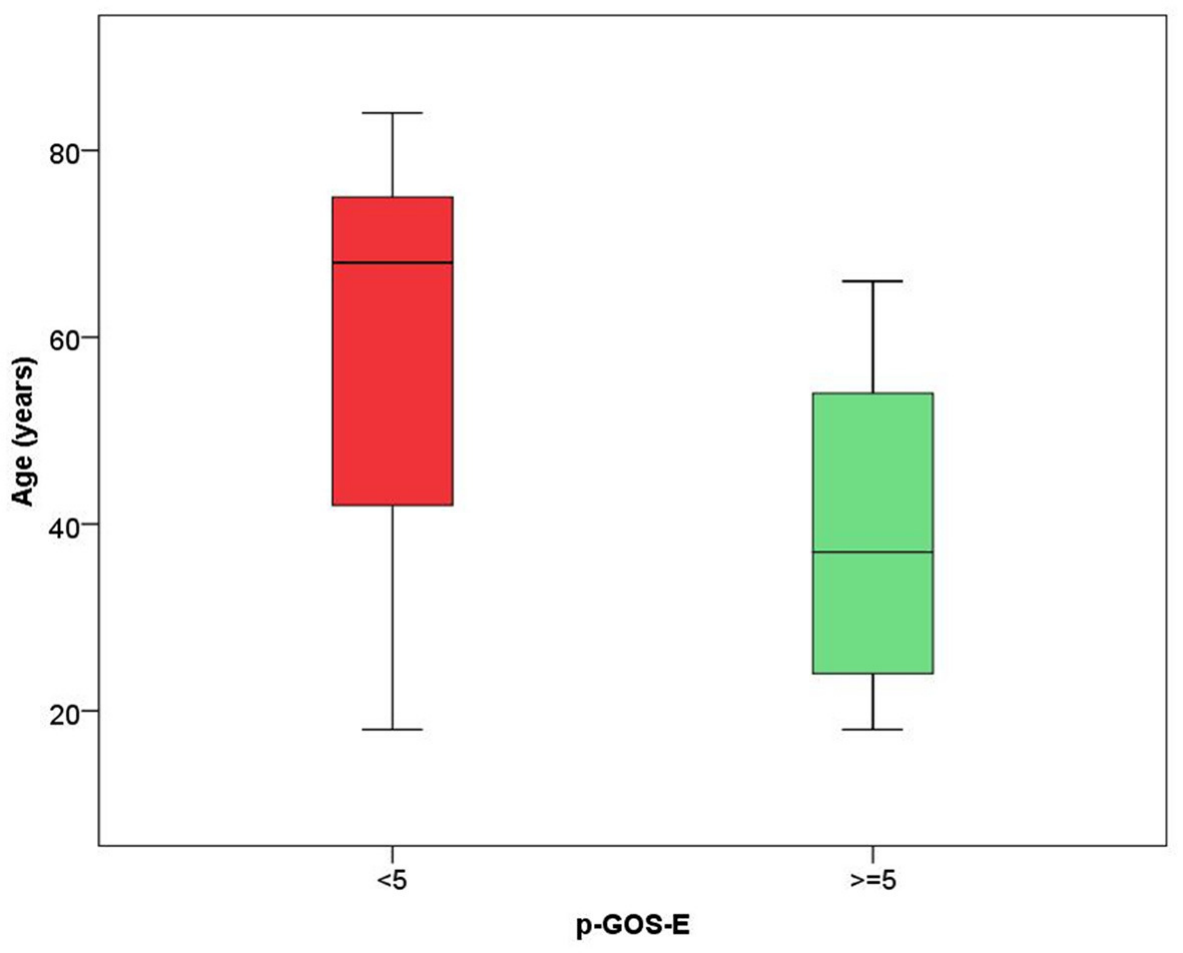
Age distribution according to good (pGOS ≥ 5) and poor (pGOS-E < 5) functional autonomy at follow-up.

**Figure 3:**
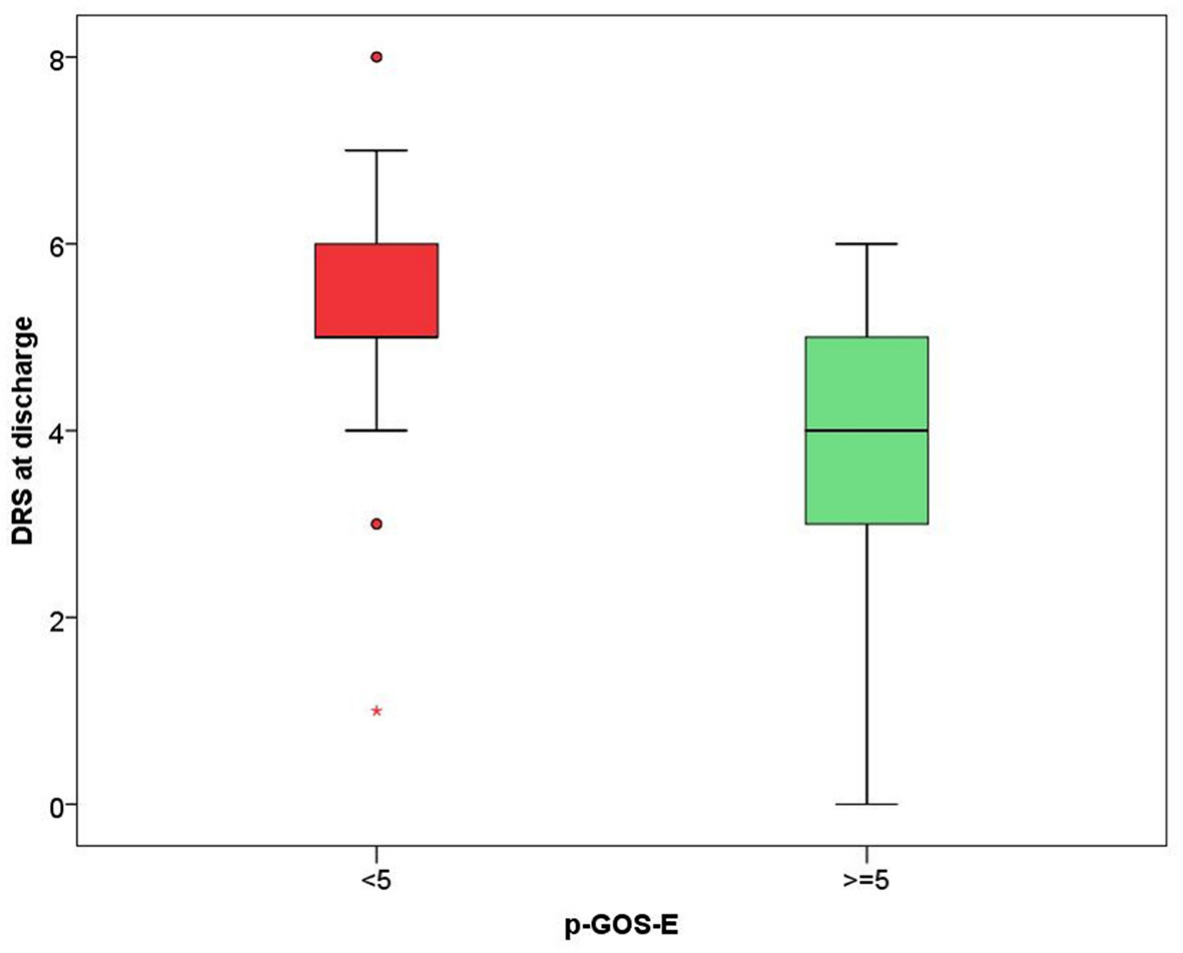
DRS score distribution according to good (pGOS ≥ 5) and poor (pGOS-E < 5) functional autonomy at follow-up.

In the model including demographic characteristics and clinical data at admission (Model A), a higher pGOS-E score at follow-up remained significantly associated only with younger age (*B* = −0.037, *p* = 0.004). In the multivariate linear regression model including demographic characteristics and clinical data at discharge (Model B), only younger age (*B* = −0.035, *p* = 0.004) and a lower DRS category at discharge (*B* = −0.392, *p* = 0.029) were significantly related to a higher functional autonomy at follow-up. In both models, the time between the event and follow-up did not achieve a statistical significance suggesting an association between age and pGOS-E as well as between DRS at discharge and pGOS-E independent of time between the event and the evaluation. In Figures 2, 3, the distribution of data significantly related to a higher long-term functional autonomy is shown. For representative purposes, the outcome variable (pGOS-E) was dichotomized to good outcome (pGOS-E ≥ 5, moderate disability or good recovery) vs. poor outcome (pGOS-E < 5, severe disability).

## 4. Discussion

In the present study, a higher CR, as measured by educational level and CRIq, was not associated with better long-term functional autonomy in survivors of sTBI. Only age and disability level at discharge from the IRU were associated with a higher functional outcome in the long term.

These results are in line with previous studies showing the absence of a protective effect of CR on the long-term neuropsychological profile of patients with sTBI (19, 21) allowing to extend these previous findings also to the long-term level of autonomy. The apparent contrast with other studies that concluded a protective effect of the CR on disability may be due to the different case-mix characteristics of the participants. First, as mentioned before, the present study, differently from other studies in this field (18, 20, 22, 23), included only patients with sTBI. Indeed, patients with sTBI are vastly different, when compared to those with moderate TBI, in terms of neurological characteristics as they include patients with DoC or confusional states but also in terms of clinical and care burden and prognosis (8). From a rehabilitation perspective, these patients are considered differently than mild and moderate TBI and are subjected to different treatment protocols (10). They, therefore, should be considered as a different group and analyzed separately to better investigate prognosis predictors (6). Second, compared to other study samples including only sTBI patients, the present sample has an older median age at injury onset: 54, IQR: 35 years; range = 18–85 compared to a mean of 26.06 years (SD = 8.2, range = 18–58) in Levi's study (38) and a mean of 26.16 years (SD = 8.8, range = 16–42) in Kesler's study (20). As shown by a recent study assessing sTBI, older age is associated with higher mortality and lower functional independence recovery (39). In addition, other results suggested that functional independence recovery was associated only with the trauma severity and the age of these patients (39).

In essence, compared to previous studies, the present analysis was carried out on older patients surviving a more severe trauma, thus with a lower brain reserve. In the reserve theory, two different models, namely the “active model” and the “passive model,” have been defined (11). The CR refers to the “active” reserve model, where the brain actively tries to cope with brain damage using pre-existing cognitive resources. So far, however, the evidence of this theory applied to traumatic brain damage is still debated, and the need for research that examines the complex interplay between a range of moderating and mediating injury-related (e.g., cause and severity of TBI) and person-related (brain reserve and CR) variables has been highlighted (16). The brain reserve, made up of a number of neuronal resources (40) and, therefore, closely related to age, represents instead the “passive” component of this reserve theory and appears to have a role in preventing cognitive decline in the advanced phases of some neurological diseases. In Alzheimer's disease, it is noteworthy that the pathology of beta-amyloid and tau accumulates decades before the cognitive impairment onset. In that sub-clinical laps of time, brain reserve could play a protective role in counteracting the effects of the disease until the disease increases up to a critical threshold in which the symptoms can no longer be contrasted. The concept of a threshold, beyond which a potential protective effect vanishes, has also been suggested in the cognitive decline associated with multiple sclerosis. Amato et al. showed that CR positively impacts cognitive performances in the early stages of illness (41), but this effect decreases with increasing levels of brain tissue loss, as measured by total brain volume and cortical volume measurements (42). Also, a recent study has shown the absence of a protective role of CR on cognitive decline in the oldest-old subjects (age > 90 years) whose brain reserve had become physiologically lower (43). From these results, it can be hypothesized that the observed protective role of the CR against traumatic brain damage vanishes after a given brain reserve decline threshold produced by both the growing patient's age and the severity of the traumatic brain injury.

The threshold model has been scarcely investigated in TBI because of the high complexity of the subsequent brain damage that often involved more than a single structural or functional area, affects a highly heterogeneous population, and may generate a diffuse axonal injury that affects the structure and function of various brain regions in ways which can be challenging to quantify reliably (44). A previous study assessing the effect of cortical atrophy as a surrogate of the brain reserve has confirmed its threshold-protective effect after an sTBI (20). The study also concluded for a protective effect of the CR in the short term after an sTBI (20); however, the population involved was very young [mean age 26.06 years (SD = 8.2, range = 18–58)], and no longitudinal follow-up was performed to explore the long-term effects of the brain damage that are known to evolve even after the occurrence of acute damage. Indeed, although TBI is an acute event, the injury triggers a cascade of neurological and psychosocial sequelae that generate a chronic disease process that may contribute to a permanent disability (9, 45).

This study has some limitations that warrant discussion. The pGOS-E, as an outcome measure of long-term functional disability, has been chosen refereeing to the minimum evaluation protocol published by the Italian society of physical and rehabilitation medicine (6). However, despite being the most widely used outcome measure after TBIs, the GOS-E is increasingly recognized to have important limitations (31). Indeed, the GOS-E, by asking patients and caregivers to evaluate a current functional state in comparison with a preinjury functionality, necessarily faces subjective biases which include patients not having full insight and caregivers overestimating the extent of a disability. This is an acknowledged limitation of the GOS-E and thus of our analysis, too. In addition, these data need to be confirmed by further studies in larger samples. Moreover, the absence of data concerning the rehabilitation treatment after the IRU discharge may represent a further limitation. Nevertheless, the primary aim of the study was to explore the impact of CR as an early predictor of the functioning of patients with sTBI admitted to the IRU and to provide information on long-term functional prognosis based on data available at admission and discharge. Overall, these results enrich knowledge about patients surviving an sTBI admitted to a rehabilitation ward and provide clinicians with more information concerning the long-term prognosis after sTBI allowing better communication with the patients and their caregivers.

## 5. Conclusion

The protective effect of CR intended as an active individual contribution based on a more stimulating lifestyle able to contrast the effects of TBI is closely related to brain reserve preservation. It is conceivable that, after an sTBI, the decline of the passive and unchangeable component of the cerebral model known as the brain reserve has a major impact on the definition of the functional outcome. Also, increasing age, in turn, further reduces the brain reserve. Future studies are needed to better investigate the subtle interaction between the brain reserve components in influencing the recovery from sTBI.

## Data availability statement

The data that support the findings of this study are available from the corresponding author [SP], upon reasonable request.

## Ethics statement

The studies involving human participants were reviewed and approved by Institutional Ethics Committee, approval number 17505_oss. The patients/participants provided their written informed consent to participate in this study.

## Author contributions

BH and FC contributed to the conception and design of the study. AR, FD, and DM organized the database. SP and AM performed the statistical analysis. BH wrote the first draft of the manuscript. SP wrote sections of the manuscript. All authors contributed to the manuscript revision and read and approved the submitted version.

## References

[1] Capizzi A Woo J Verduzco-Gutierrez M. Traumatic brain injury. Med Clin North Am. (2020) 104:213–38. 10.1016/j.mcna.2019.11.001 32035565

[2] Tagliaferri F Compagnone C Korsic M Servadei F Kraus J. A systematic review of brain injury epidemiology in Europe. Acta Neurochir. (2006) 148:255–68. 10.1007/s00701-005-0651-y 16311842

[3] Millis SR Rosenthal M Novack TA Sherer M Nick TG Kreutzer JS. Long-term neuropsychological outcome after traumatic brain injury. J Head Trauma Rehabil. (2001) 16:343–55. 10.1097/00001199-200108000-00005 11461657

[4] Collaborators MCT. Predicting outcome after traumatic brain injury: practical prognostic models based on large cohort of international patients. BMJ. (2008) 336:425–9. 10.1136/bmj.39461.643438.25 18270239 PMC2249681

[5] Murray GD Butcher I McHugh GS Lu J Mushkudiani NA Maas AIR. Multivariable prognostic analysis in traumatic brain injury: results from the IMPACT study. J Neurotrauma. (2007) 24:329–37. 10.1089/neu.2006.0035 17375997

[6] Lavezzi S Bargellesi S Cassio A De Tanti A Gatta G Hakiki B. Redefining a minimal rehabilitation assessment protocol for severe acquired brain injuries. Eur J Phys Rehabil Med. (2022) 58:584–91. 10.23736/S1973-9087.22.07451-2 35666492 PMC9980564

[7] Edlow BL Claassen J Schiff ND Greer DM. Recovery from disorders of consciousness: mechanisms, prognosis and emerging therapies. Nat Rev Neurol. (2021) 17:135–56. 10.1038/s41582-020-00428-x 33318675 PMC7734616

[8] Kowalski RG Hammond FM Weintraub AH Nakase-Richardson R Zafonte RD Whyte J. Recovery of consciousness and functional outcome in moderate and severe traumatic brain injury. JAMA Neurol. (2021) 78:548. 10.1001/jamaneurol.2021.0084 33646273 PMC7922241

[9] LoBue C Munro C Schaffert J Didehbani N Hart J Batjer H. Traumatic brain injury and risk of long-term brain changes, accumulation of pathological markers, and developing dementia: a review. J Alzheimers Dis. (2019) 70:629–54. 10.3233/JAD-190028 31282414

[10] Moretti L Cristofori I Weaver SM Chau A Portelli JN Grafman J. Cognitive decline in older adults with a history of traumatic brain injury. Lancet Neurol. (2012) 11:1103–12. 10.1016/S1474-4422(12)70226-0 23153408

[11] Jennett B Bond M. Assessment of outcome after severe brain damage. Lancet Lond Engl. (1975) 1:480–4. 46957 10.1016/s0140-6736(75)92830-5

[12] Stern Y. What is cognitive reserve? Theory and research application of the reserve concept. J Int Neuropsychol Soc. (2002) 8:448–60. 10.1017/S1355617702813248 11939702

[13] Stern Y. Cognitive reserve in ageing and Alzheimer's disease. Lancet Neurol. (2012) 11:1006–12. 10.1016/S1474-4422(12)70191-6 23079557 PMC3507991

[14] Stern Y. Cognitive reserve. Neuropsychologia. (2009) 47:2015–28. 10.1016/j.neuropsychologia.2009.03.004 19467352 PMC2739591

[15] Murray AD Staff RT McNeil CJ Salarirad S Ahearn TS Mustafa N. The balance between cognitive reserve and brain imaging biomarkers of cerebrovascular and Alzheimer's diseases. Brain J Neurol. (2011) 134(Pt 12):3687–96. 10.1093/brain/awr259 22102649

[16] Mathias JL Wheaton P. Contribution of brain or biological reserve and cognitive or neural reserve to outcome after TBI: a meta-analysis (prior to 2015). Neurosci Biobehav Rev. (2015) 55:573–93. 10.1016/j.neubiorev.2015.06.001 26054792

[17] Rentz DM Locascio JJ Becker JA Moran EK Eng E Buckner RL. Cognition, reserve, and amyloid deposition in normal aging. Ann Neurol. (2010) 67:353–64. 10.1002/ana.21904 20373347 PMC3074985

[18] Chang L Tomasi D Yakupov R Lozar C Arnold S Caparelli E. Adaptation of the attention network in human immunodeficiency virus brain injury. Ann Neurol. (2004) 56:259–72. 10.1002/ana.20190 15293278

[19] Sumowski JF Wylie GR Gonnella A Chiaravalloti N DeLuca J. Premorbid cognitive leisure independently contributes to cognitive reserve in multiple sclerosis. Neurology. (2010) 75:1428–31. 10.1212/WNL.0b013e3181f881a6 20956787 PMC3039206

[20] Kesler SR Adams HF Blasey CM Bigler ED. Premorbid intellectual functioning, education, and brain size in traumatic brain injury: an investigation of the cognitive reserve hypothesis. Appl Neuropsychol. (2003) 10:153–62. 10.1207/S15324826AN1003_04 12890641

[21] Fuentes A McKay C Hay C. Cognitive reserve in paediatric traumatic brain injury: relationship with neuropsychological outcome. Brain Inj. (2010) 24:995–1002. 10.3109/02699052.2010.489791 20515361

[22] Schneider EB Sur S Raymont V Duckworth J Kowalski RG Efron DT. Functional recovery after moderate/severe traumatic brain injury: a role for cognitive reserve? Neurology. (2014) 82:1636–42. 10.1212/WNL.0000000000000379 24759845 PMC4211893

[23] Steward KA Kennedy R Novack TA Crowe M Marson DC Triebel KL. The role of cognitive reserve in recovery from traumatic brain injury. J Head Trauma Rehabil. (2018) 33:E18. 10.1097/HTR.0000000000000325 28520675 PMC5693786

[24] Fraser EE Downing MG Biernacki K McKenzie DP Ponsford JL. Cognitive reserve and age predict cognitive recovery after mild to severe traumatic brain injury. J Neurotrauma. (2019) 36:2753–61. 10.1089/neu.2019.6430 31017049

[25] Stenberg J Håberg AK Follestad T Olsen A Iverson GL Terry DP. Cognitive reserve moderates cognitive outcome after mild traumatic brain injury. Arch Phys Med Rehabil. (2020) 101:72–80. 10.1016/j.apmr.2019.08.477 31562876

[26] Leary JB Kim GY Bradley CL Hussain UZ Sacco M Bernad M. The association of cognitive reserve in chronic-phase functional and neuropsychological outcomes following traumatic brain injury. J Head Trauma Rehabil. (2018) 33:E28. 10.1097/HTR.0000000000000329 28731870 PMC5752441

[27] Jeon IC Kim OL Kim MS Kim SH Chang CH Bai DS. The effect of premorbid demographic factors on the recovery of neurocognitive function in traumatic brain injury patients. J Korean Neurosurg Soc. (2008) 44:295. 10.3340/jkns.2008.44.5.295 19119465 PMC2612566

[28] Bertoni D Petraglia F Basagni B Pedrazzi G De Gaetano K Costantino C. Cognitive reserve index and functional and cognitive outcomes in severe acquired brain injury: a pilot study. Appl Neuropsychol Adult. (2020) 2020:1–11. 10.1080/23279095.2020.1804910 32795208

[29] von Elm E Altman DG Egger M Pocock SJ Gøtzsche PC Vandenbroucke JP. The strengthening the reporting of observational studies in epidemiology (STROBE) statement: guidelines for reporting observational studies. Bull World Health Organ. (2007) 85:867–72. 10.1016/j.jclinepi.2007.11.008 18038077 PMC2636253

[30] Levin HS Boake C Song J McCauley S Contant C Diaz-Marchan P. Validity and sensitivity to change of the extended glasgow outcome scale in mild to moderate traumatic brain injury. J Neurotrauma. (2001) 18:575–84. 10.1089/089771501750291819 11437080

[31] Wilson JTL Edwards P Fiddes H Stewart E Teasdale GM. Reliability of postal questionnaires for the glasgow outcome scale. J Neurotrauma. (2002) 19:999–1005. 10.1089/089771502760341910 12482113

[32] Lombardi F Gatta G Sacco S Muratori A Carolei A. The Italian version of the coma recovery scale-revised (CRS-R). Funct Neurol. (2007) 22:47–61. 17509244

[33] Hagen C Malkmus D Durham P. Cognitive assessment and goal setting. In: Rehabilitation of the Head Injured Adult: Comprehensive Management. Downey Ca: Rancho Los Amigos Hospital Inc. (1979)

[34] Rappaport M Hall KM Hopkins K Belleza T Cope DN. Disability rating scale for severe head trauma: coma to community. Arch Phys Med Rehabil. (1982) 63:118–23. 7073452

[35] Levin HS O'Donnell VM Grossman RG. The galveston orientation and amnesia test: a practical scale to assess cognition after head injury. J Nerv Ment Dis. (1979) 167:675–84. 501342 10.1097/00005053-197911000-00004

[36] Jennett B Snoek J Bond MR Brooks N. Disability after severe head injury: observations on the use of the Glasgow Outcome Scale. J Neurol Neurosurg Psychiatry. (1981) 44:285–93. 6453957 10.1136/jnnp.44.4.285 PMC490949

[37] Mancuso M Varalta V Sardella L Capitani D Zoccolotti P Antonucci G. Italian normative data for a stroke specific cognitive screening tool: the Oxford Cognitive Screen (OCS). Neurol Sci. (2016) 37:1713–21. 10.1007/s10072-016-2650-6 27395388

[38] Levi Y Rassovsky Y Agranov E Sela-Kaufman M Vakil E. Cognitive reserve components as expressed in traumatic brain injury. J Int Neuropsychol Soc. (2013) 19:664–71. 10.1017/S1355617713000192 23575273

[39] Maiden MJ Cameron PA Rosenfeld JV Cooper DJ McLellan S Gabbe BJ. Long-term outcomes after severe traumatic brain injury in older adults. A registry-based cohort study. Am J Respir Crit Care Med. (2020) 201:167–77. 10.1164/rccm.201903-0673OC 31657946

[40] Stern Y. An approach to studying the neural correlates of reserve. Brain Imag Behav. (2017) 11:410–6. 10.1007/s11682-016-9566-x 27450378 PMC5810375

[41] Sumowski JF Chiaravalloti N Wylie G Deluca J. Cognitive reserve moderates the negative effect of brain atrophy on cognitive efficiency in multiple sclerosis. J Int Neuropsychol Soc. (2009) 15:606–12. 10.1017/S1355617709090912 19573279

[42] Amato MP Razzolini L Goretti B Stromillo ML Rossi F Giorgio A. Cognitive reserve and cortical atrophy in multiple sclerosis: a longitudinal study. Neurology. (2013) 80:1728–33. 10.1212/WNL.0b013e3182918c6f 23576622

[43] Hakiki B Pancani S Portaccio E Molino-Lova R Sofi F Macchi C. Impact of occupational complexity on cognitive decline in the oldest-old. Aging Ment Health. (2021) 25:1630–5. 10.1080/13607863.2020.1746739 32252551

[44] Irimia A Goh SY Torgerson CM Vespa PM Van Horn JD. Structural and connectomic neuroimaging for the personalized study of longitudinal alterations in cortical shape, thickness and connectivity after traumatic brain injury. J Neurosurg Sci. (2014) 58:129. 24844173 PMC4158854

[45] Corrigan JD Cuthbert JP Harrison-Felix C Whiteneck GG Bell JM Miller AC. US population estimates of health and social outcomes 5 years after rehabilitation for traumatic brain injury. J Head Trauma Rehabil. (2014) 29:E1–9. 10.1097/HTR.0000000000000020 24495919

